# Multiple perspectives and dialogue in understanding experiences of living with eating disorders: Two narratives—four unpackings

**DOI:** 10.1186/s40337-022-00554-5

**Published:** 2022-02-15

**Authors:** Berit Støre Brinchmann, Siri Lyngmo, Sine Maria Herholdt-Lomholdt, Bodil H. Blix

**Affiliations:** 1 The Faculty of Nursing and Health Sciences, Nord University, 8026 Bodø, Norway; 2 Regional Centre for Eating Disorders, Nordland Hospital, 8076 Bodø, Norway; 3 Department of Health and Care Sciences, Faculty of Health Sciences, UiT The Arctic University of Norway, 9037 Tromsø, Norway

**Keywords:** Dialogue, Eating disorder, Metaphor, Multiple perspectives, Narrative

## Abstract

**Background:**

This is a response to Conti et al.’s article, “Listening in the dark: why we need stories of people living with severe and enduring anorexia nervosa” (published in JED, 2016), and its call for relational metaphors and a relational approach to supplement the traditional medical/psychological diagnostic language used to describe the life experiences and complex emotions of people affected by an eating disorder.

**Methods:**

Four authors with different backgrounds unpack two narratives, ‘*The Prima Donna with the Green Dress*’ and ‘*Breaking down the Wall*’, both narrated during fieldwork in multifamily therapy. The narratives are unpacked from the perspective of a therapist within multifamily therapy, a researcher who conducted the fieldwork, a researcher based in phenomenology and a researcher based in narrative inquiry. The authors enter into dialogue with the narratives, and with each other.

**Results:**

The four authors focus on different elements within the narratives and understand them differently. One, focuses on strength and pride, and art expression as a different form of language for people living with an eating disorder. Another, on the experience of isolation, boundaries, and balancing openness and closedness. A third, sees the narratives as expressing a wish to see and be seen, and the fourth focuses on the absence of, and longing for, a shared space to explore.

**Conclusion:**

The aim is not to reach a correct or shared interpretation of the narratives but to explore how different perspectives may contribute to different insights, not only about one family in particular but about, more generally, the experiences of people living with an eating disorder. Our work shows the significance of engaging with multiple perspectives and dialogue as supplements to the traditional medical/psychiatric diagnostic language in both clinical practice and research.

## Plain English summary

In this article, four authors read and unpack two narratives from fieldwork in a multifamily therapy programme for adults with a severe eating disorder. The authors have different professional, theoretical and methodological backgrounds. The aim is not to reach a shared understanding of the narratives, but rather to explore how different perspectives contribute to different insights. Multiple perspectives and dialogue may shed light on people’s experiences of living with a severe eating disorder in both clinical practice and research.

## Introduction

This article is a response to the article “Listening in the dark: why we need stories of people living with severe and enduring anorexia nervosa” [1], and its appeal for other perspectives resonating better with the lived experience of those whose treatment has failed. We concur with the authors’ statement, “Beyond the randomized trials and manuals it is time for us to listen to the realities of suffering, the minutia of resistance and the life that can still be lived” [1, p. 1]. This article is based on two narratives from fieldwork in a multifamily therapy (MFT) programme for young adults suffering a severe eating disorder (ED) [2–5]. The four authors, Siri, a nurse and MFT therapist, Berit a nurse and researcher who conducted fieldwork in the MFT programme, Sine a nurse and researcher based in phenomenology, and Bodil a nurse and researcher based in narrative inquiry, unpack the two narratives from our respective perspectives. Our aim is to expand the understanding of the experience of living with an ED, and further, to reflect on the potential for engaging with such experiences through different perspectives. This article is based on two narratives: ‘The Prima Donna with the Green Dress’ and ‘Breaking Down the Wall’.

## Background

EDs are serious mental disorders that impact many aspects of people’s lives [6]. EDs are, also, the mental illnesses with the highest mortality [7].

A systematic review looking at the relationship between non-abusive adverse life experiences and EDs found these difficulties, in home and family environments, to be more prevalent in relation to bulimia nervosa (BN) and binge-eating disorder than with anorexia nervosa (AN) [8]. Research suggests that individuals with an ED are relatively likely to have been abused or neglected as children, or to have been victimised in adolescence or adulthood. These experiences, in turn, are often associated with a range of psychological symptoms, as well as, in some cases, a more severe or complex ED presentation [9–13]. Previous studies have pointed to the complex relationship between post-traumatic stress, borderline personality disorder and EDs [14], and PTSD, depressive symptoms and disordered eating following sexual assault [15]. In a recent qualitative study [16], trauma-exposed inpatients were asked about their experience of the impact of traumatic stress on their eating behaviour. The findings showed that they experienced eating behaviour as a coping strategy, and that feelings of stress governed eating behaviour. One often sees that eating behaviour keeps symptom pressure away from underlying and possibly unconscious problems, and when patients begin to eat and regain normal weight, these symptoms can re-emerge with or without conscious awareness of them. The ED often serves to carry the family’s symptoms, whilst the core of the problem may lie elsewhere.

Despite the serious nature of the disorder, research has shown that those with an ED can have an ambivalent relationship to it, with both negative and positive feelings. In a recent metasynthesis of adult AN treatment experiences [17], several physical problems were reported. However, across all the included studies, the participants described ambivalence in relation to the question of whether, or not, AN was problematic, including a sense of pride, achievement and control that was hinged upon the thin body. A qualitative study of women with severe enduring AN (SEED-AN) [18] described feelings of unworthiness, depression and hopelessness but also of having achieved a sense of pride in having endured and survived despite the ED. A qualitative synthesis of AN’s meaning to patients [19] mentions positive aspects of the disease such as feelings of power, being special, superior, beautiful, popular and attractive, and as a way of gaining attention. Patients with AN may experience ‘mental strength’ (inner sense of mastery) and ‘self- confidence’ (feeling acknowledged and worthy of compliments) [20]. We have also found several studies concerning pride and shame among women with an ED [21–23] and pride among women with an ED [24].

Research shows that EDs greatly affect parents, siblings and the wider family [25–29] and are experienced as more demanding for the family than, for example, psychotic disorders [30–32]. Furthermore, studies show that those closest to a family member with an ED suffer from isolation and receive little help from support services [27–29, 33–35].

Two earlier articles presented the results of fieldwork in an MFT group for adults with an ED [2, 3]. The studies considered the patients’ and their families’ experience of MFT [2] as well as the experiences of the therapists involved [3]. The implementation of MFT for adults with an ED has been presented in some detail earlier [2, 4, 5], and will, in this article, be described only briefly as context for this study.

### Study context

Since 2007, an MFT model for an adjunct psychotherapeutic approach to the treatment of young adult patients (aged 18–30) suffering from a severe ED has been continuously developed at the Regional Centre for Eating Disorders (RESSP) at Nordland Hospital in Bodø, Norway. The MFT includes family members and involves the participation of six to eight families. These—young adult ED patients and their families—take part in a diverse model of MFT, previously over a twelve-month period. Rather than challenging the ED directly, the MFT aims to support the taking of adult responsibility and the encouragement of, and support for, better communication and interaction within the families [2, 4, 5]. MFT employs a number of group settings—plenary groups of patients and families and smaller groups of mothers, fathers, siblings or patients, and a range of interventions including role-play, family mapping and sculpts and other creative work. Important themes such as relationships, communication, conflict management, interactivity, motivation, and feelings of guilt are addressed in both psycho-educational and clinical groupwork settings [2, 3]. The MFT draws on several theoretical models; psychodrama, systemic family therapy, collaborative therapy, mentalising-based therapy, mindfulness, multifamily therapy for psychosis and cognitive therapy. In addition, a number of exercises have been developed by the RESSP team [2–5].

Kvebæk developed a family sculpture technique [36], where family members placed figures to demonstrate how they saw their relationships now, and how they wanted them to be. He then talked with families about the implications of their different points of view. Kvebæk saw figure placements as projections of an inner reality. Balmbra [37], who has been one of those developing MFT at RESSP, regards them, however, as an invitation to dialogue, rather than a means of assessment. Figures are employed as a visual means of expression in ongoing dialogue, and this has its roots in family sculpture, collaborative and narrative approaches, as well as the problem system approach [37]. “Sculpting our family with figures,” [5] (pp. 194–195) is one of the exercises included in the MFT. The exercise is about bringing the family together to gain a clearer idea of each other’s views, to express fears for the future, and then looking at how they each can contribute to improve the situation. The families go to separate rooms together with a therapist and sit around a table. Each family member is given a Playmobil figure to represent themselves, and a model of the ED, made by the young person with an ED. Each family member is asked to arrange the figures to show how they see their family at present, how they worry that things might turn out, and what they want for their family in the future, using positions, closeness and distance. The idea is that seeing the figures arranged in relation to each other will give a unique perspective on the family that can bring out fresh ideas, and that comparing similarities and differences in the perspectives can promote tolerance and the ability to mentalise [5, pp. 194–195].

## Methods

The data comprised approximately one hundred and eighty hours of field observations in two MFT groups. Twelve young women (18–22 years, eight with AN and four with BN), twelve sets of parents, eight sisters, one brother, one grandmother and two partners participated in the two groups. Most patients in the groups received treatment in community-based clinics, but some were inpatients [2]. The fieldwork, conducted by Berit between 2015 and 2017, included participation in planning, ongoing evaluation, supervision meetings for the therapists, and field observations in MFT groups. Moreover, Berit conducted individual qualitative interviews with two patients, twelve family members, eight therapists, and group interviews with seven patients and eighteen family members. In the groups, Berit was present as an ‘ordinary participant’, taking part in activities on more or less the same basis as the other participants, while writing contemporaneous field notes.

This article is based on two narratives—one from Berit’s fieldnotes from the participant observation of the MFT programme exercise ‘Sculpting our families with figures’ (‘The Prima Donna with the Green Dress’), and one from Berit’s interview with one of the fathers in the MFT programme (‘Breaking Down the Wall’). Both narratives involve the same family. The two narratives were strategically selected for further inquiry and discussion because they made a particular impression on Berit.

### Ethics

The study conforms to the principles outlined in the Declaration of Helsinki. The research project was approved by Regional Committee for Medical and Health Research Ethics (REK; reference 2014/1621/REK vest). In addition, all participants and therapists in the MFT groups received verbal and written information regarding the research project and signed consent forms. The data were treated confidentially and anonymised. After the fieldwork was completed, Berit contacted the family who was involved in the two narratives with an invitation to participate in further research. The family declined this invitation. Out of respect for this decision, we did not contact the family while working on this publication.

### Analysis

In the following, we present the two narratives: ‘*The Prima Donna with the Green Dress*’ and ‘*Breaking Down the Wall*’. The first narrative is based on fieldnotes from the MFT exercise “Sculpting our family with figures”. The second narrative is based on an individual interview with one of the fathers in the MFT programme.

Following the two narratives, we present our respective unpackings of the narratives. From the perspective of the MFT therapist, Siri unpacks the narrative ‘*The Prima Donna with the Green Dress*’. Siri’s unpacking was audiotaped and transcribed by Berit who, on the basis of this, wrote a draft of Siri’s unpacking which was discussed and finalised in collaboration with Siri. Berit unpacks the two narratives from the field researchers’ perspective. Sine’s phenomenological unpacking of the two narratives follows. Finally, Bodil unpacks the two narratives from the perspective of the narrative inquirer. The analysis was a dialogical process in which we read and discussed each other’s texts and rewrote our individual unpackings based on the feedback from the other authors, as the process moved forward. The aim of this process was not to reach a common understanding of the two narratives, to find the ‘true interpretation’, so to speak. Rather, we strived to learn from each other’s unpackings to reach a deeper and more nuanced understanding of the complex experience of living with an ED.

According to Gadamer [38], understanding the human world is always interpretations based in cultures, traditions, and historical epochs. Richer and deeper understandings are reached when different horizons of understandings come into dialogue. Understandings are always drafts, continuously and always under revision. Understandings are, in Gadamer’s sense, always pre-liminary. Gadamer’s perspective on understanding resonates with Geertz’s [39] anthropological conceptualization of ‘thick description’ which, he argued, is an ongoing process of interpretation always open to further interpretation. As Geertz writes, his analysis of culture is not “in search of law but an interpretive one in search for meaning” [39, p. 5]. And such analysis is a search for possible structures of signification [39, p. 9], with the aim of enlargement of “the universe of human discourse” [39, p. 14]. Moreover, there is a close connection between thick description and narrative [40]. In research, it is impossible to tell “the whole story”, but we nonetheless strive to give voice to participants’ stories, “but always mediated by the interpretive lens brought to the telling by the researcher and the circumstances in which the research is being carried out” [40].

## Results

### Two narratives

#### The Prima Donna with the green dress

The young women were given a task of making a small sculpture of their ED experience out of a Playmobil skeleton figure and plasticine. One of them had dressed the skeleton in a beautiful floor-length green dress, edged with a broad border of flames. In her family group, the young woman described the ED as the family’s centre point and Prima Donna. Her parents, brother and sister reflected on the ED’s large place in the family, and what they could do to change their troubled situation. “I know what has to be done” the young woman said, “but I am the only one with the key to the solution.” (Fieldnote, MFT group exercise).

#### Breaking down the wall

Before we started in MFT it was as if there was a wall between her and us in relation to the disease, but then something happened in the MFT which meant that the wall was broken down a bit. We knew what we could talk about, had some shared experiences around the illness that we could speak about—because we have been so scared—am I crossing a boundary here if I say? …. or is it something I mustn’t say? You become very watchful.

This wall really set a limit in that she didn’t really know what to say to us, because we didn’t really understand what this was all about. And so, you sit on the other side of the wall, afraid that if you say this or that it might be wrong, or, there again, maybe smart. You sit and wonder what to do: Should you try to tempt her with something? Should she get something if she does it? You sit there like a question mark. And you don’t start to climb the wall because you don’t know what awaits you on the other side…” (Interview, father) [2, p. 7].

### Four unpackings

#### Siri

Siri is an experienced MFT therapist. She has extensive experience of working with adults with an ED as an individual, family and group therapist and has been part of the development of MFT at RESSP [5]. She is a trained, clinically specialised, psychiatric nurse, and also has training in supervision, systemic family therapy, psychodrama, expressive art therapy, body therapy, yoga and mindfulness.

I remember the exercise with this family very well. In addition to the woman with an ED, there was a brother and a sister, a Mum and a Dad. Each member of the family was to present the others (using Playmobil figures) in terms of proximity and distance. The arrangement was a very strong picture with lots of symbolism. Symbolism does not always provide an answer, but it may provide us with another kind of language: both the positioning and disposition of the figures with regard to closeness and distance, but also the self-presentation of the young woman in the sculpture—the fantastic long green dress with its flames in yellow, orange, red and blue all around them, and also her long hair. Hair may represent feelings.

In my unpacking I am interpreting the image through the lens of the chakra system. This is one way of a range of interpretations of the young woman’s choice to depict herself wearing a green dress and how she situated herself in relation to her family with the Playmobil figures. As a part of the chakra system, colours are symbolic. The chakra system is outside the traditional (medical and psychological) way of thinking, but it has a place both in art and expressive therapy, psychodrama, body therapy and yoga therapy and represents an ancient tradition presented in several religions and cultures.

Towards the end of the nineteenth-century, the Theosophists started to take an interest in Eastern metaphysics. A number of books were published, including books about the chakra system, among these, ‘The Chakras’ by C. W. Leadbeater [41]. The word ‘chakra’ is Sanskrit and means ‘wheel’ or ‘wheel of life’. The chakras are subtle energy centres for the exchange of energy between people and their surroundings and are described as concentrated energy vortices. The chakra system is what and who we are, what we feel, how we feel and how we change. Just as we have a physical body, we also have a subtle body, an energetic body. The chakras form a bridge between our materiality and the subtle substance which is a part of us. The chakras are also connected to each other and affect the endocrine system, and thereby have a considerable influence on our mental and physical well-being. Each chakra has a specific color. These follow each other in the same series as the rainbow: red, orange, yellow, green, blue, indigo and violet [42].

Perhaps the dress with the flames was not only an expression of the ED, but also represented *her* (the young woman with an ED)? The three lowest chakras have to do with being firmly grounded, with being present on earth, sexuality and creativity, and the third chakra concerns feelings. The blue is associated with communication, the green with heart and compassion (and all the good things we associate with them). This makes me wonder if it is burning around something that has to do with her lower chakras, that is, issues of identity, creativity and sexuality? Is there something she cannot convey?

The Playmobil figure showed an upright, proud, very elegant woman, with the green dress with flames touching the ground, like a real Prima Donna. I wonder if the young woman was teasing the others in the family by saying that only she had the key to the solution? Did she tell them that she was not going to give the ED up at any price? This pride—it could look like she was very powerful and wanted to keep the others on tenterhooks to get what she wanted. I wonder if the Playmobil figure represented herself as the tough one and the rest of the family as puppets ready to fulfill her needs? Or did the figure symbolise that she was the strong one, carrying the family history/secrets, and protecting them from a ‘revelation’? Could it be that the young woman had tried to say things, in her own way, without being heard in the way she needed? Maybe the Playmobil figure exercise provided the young woman an opportunity to convey something through a different type of language. It is not always possible to say things in words, and other forms of expression are used instead. Some experiences may be in the body at a pre-linguistic level.

Another approach, within art and expressive therapy, is to imagine a triangle comprising the therapist, patient and a particular artefact—e.g., a painting. For some people, it is easier and less threatening to talk about and reflect on something outside of themselves. Playmobil figures, pictures and collage may provide the participants with opportunities to express experiences that would be difficult to express if they were to sit and talk. Art therapy and psychodrama can be enriching because they represent a language different from the academic and medical language with its talk of symptoms and diagnoses [1].

Art therapy interpretations construct one version of the person’s reality that can be understood in light of other versions, including medical and psychological knowledge. One can reflect, as a means of trying to understand what is going on, without this yielding anything concrete. One cannot reach conclusions, but this is another kind of language. The complex lived experiences of living with an ED may be expressed more comprehensively through metaphor. Moreover, metaphors may depict and give meaning to a comprehensive social reality, thereby enabling rich understandings of the lived experience of an ED and potential therapeutic opportunities. The key metaphor (“I am the only one with the key to the solution”) may be an expression of hope and agency despite the impact on the ED on her life. One can reflect on the size of keys, large and small keys, what are they used for? If the key is to work, there needs to be a keyhole. She can say, for example: “I have the key to the solution, but maybe someone else in the family has the keyhole or the right code.” There are many ways of philosophising and reflecting about such things.

#### Berit

Berit is a nurse and holds a PhD in nursing. She works as a professor and researcher at Nord University and RESSP at Nordland Hospital. She has conducted two years of fieldwork with MFT for adults with EDs and has published two articles based on this work [2, 3]. She has also supervised master’s students [27] and a PhD student [28, 29] on studies about EDs. Although she has not worked clinically with persons with EDs, she has expertise in ethics and her thinking is inspired by the Danish philosopher and theologian K.E. Løgstrup [43, 44].

I find the narrative about the wall to be a beautiful and powerful metaphor, speaking on many levels of the father’s and family’s experience of loneliness, isolation and struggles to move forward. In a previous article [2], we have described how participants in MFT experienced being helped to break down this wall, both within the family and more broadly after the MFT was completed. Breaking down the wall a little led to openness and better understanding, meaning that the young persons and their families could be better supported, by the other families but also by the support services and others.

Maybe the narrative about the wall also concerns the possibility of having a kind of wall inside, so that you don’t dare to feel or to open up about things that seem difficult or threatening. There may be layer upon layer, and several walls. One must open up to be met with understanding, but without exposing oneself. It may seem easy to talk about openness. I know from my own experience with the people I am closest to and as a friend of people with severe psychological problems, that openness is not simple in practice. If the person who suffers doesn’t want openness, what then should their nearest and dearest do? There remains a great deal of stigma attached to mental illness; and much is difficult for outsiders to understand, because problems and challenges experienced by persons with mental illness may not be apparent from the outside.

I believe that the Danish theologian and philosopher Knud E. Løgstrup [43] has written wisely on what he referred to as the zone of untouchability. Each person must lay claim to a zone of untouchability—an innermost 'space' that is surrounded by a silent 'stop'—a space which no-one may enter unless they have been invited. In this space, important things are formed in us: it is where good feelings develop, and where we work with those experiences we have no words for. It is to this place we withdraw when the clamour around us becomes all too much. Respect for this untouchability applies both inwards and outwards: inwards to protect our own needs, outwards as a boundary to the other. Usually, each of us respects the other’s zone of untouchability. When this zone is not respected, relations will be affected negatively [43]. The zone of untouchability concerns one’s integrity. If the zone of untouchability is not balanced with the need for openness to the other, the person becomes locked in. But if we fail to acknowledge and protect the zone of untouchability, our own as well as that of others’, we are in danger of violating each other. In other words, there is a limit to openness. One must protect one’s selfhood, and we all have a duty not to invade or violate the other’s zone of untouchability and integrity.

All people have a need for boundaries, in order to protect themselves. It is important to try to sense where these boundaries are. Sometimes we may have to take a step back: ‘You may come this close, but not closer—this is mine, and I don’t want to discuss it. Maybe I will talk with someone, but not with everyone, and maybe I will be open after some months when I feel more secure’.

I wonder if the narratives about the Prima Donna and the wall revolve around the same issues. As for the wall, the dress with its flaming border helps maintain a distance. No one dares to cross the flames and come close. However, if the family was able to discover how to carefully extinguish the flames and turn them into embers, they might find a way to come closer. It would not even be necessary to extinguish the flames completely, so the young woman could still feel protected. The family could, perhaps, even have a little fun, and enjoy themselves together, because there is a lot of power and heat in these flames. The same applies to the wall. The wall might be high, but lower in some places. It might be thick and sturdy, but there may also be cracks in it. Perhaps is it possible to find or make an opening in the wall to be able to start a dialogue? Is there a keyhole that matches the key, a padlock somewhere, or a code to open the wall? To get the help and understanding of others, the wall must be broken down a little, but in a way and at a pace that does not invade the young woman’s zone of untouchability or compromise her and her family’s integrity.

Family members of women with an ED are often unsure and afraid. They may focus on finding ‘the solution’ rather than on being open, questioning and reflective in looking for possible solutions together. The fear and dread that the woman will die, may obscure what would have been a better response. This may make the family members withdraw or become invasive. And gradually, the wall grows. Family members may need help to see themselves from the outside to become aware of how they affect each other. They may have to learn new ways of communicating. Some may find it difficult to be open, when feeling isolated and having lost friends and family. Some have experienced being met with facile solutions and feel misunderstood. They may have tried almost everything to make the young woman eat. Having a problem in the family is often shame laden, and the problem is often kept inside the family. However, when opening up about problems, one often gets stories in return. Everyone has experienced challenges at some point in their lives.

All people depend on each other. The dependence and vulnerability increase when something is at stake, such as when a family member becomes seriously ill. Our lives are intertwined [44]. I am entwined in your fate, and you in mine, we must relate to each other and cannot be indifferent to each other. Løgstrup [44] argues that the ground state of human life is interdependence and dependence. “…we are to a large extent inescapably dependent upon one another so that, whether the thing at stake is our mood or our destiny, we are mutually and in a most immediate sense in one another’s power. Out of this basic dependence and direct power arises the demand that we take care of that in the other person’s life which is dependent on us and which we have in our power” [44, p. 28].

#### Sine

Sine is a nurse and holds a Ph.D. in nursing aesthetics. She works as an associate professor at Nord University. Sine’s expertise is in phenomenological unpacking of descriptions of lived experience, and in using phenomenological philosophy as well as artwork and literature in such unpacking. She has neither clinical experience nor research experience with EDs.

Reading the narrative about the ‘Prima Donna with the Green Dress**’**, one certain sentence makes a particular impression on me. It is the sentence: “*the young woman described the ED as the family’s centre point and Prima Donna”.* I wonder if the beautiful green dress could be understood as the ED. The dress draws everyone’s attention; it makes the one carrying it visible. The dress is eyecatching and, as the ED, the dress might be the family’s midpoint. The dress is beautiful—but also dramatic, with red flames burning. It is alluring and frightening at the same time. I wonder how this family’s situation would be if the dress disappeared. Do they have anything in common? It seems as if the green dress gathers the family. Maybe it is not just a midpoint—maybe this dress is responsible for the coherence of the family. I wonder if there would be any kind of togetherness in this family without the dress? At the same time, I get a sense that the dress makes a silent cry: *Watch me*, it says. But sadly, *the dress* may be the only thing everyone sees. Maybe no one knows anymore who is underneath this dress. Maybe no one ever did. Maybe the one who carries the dress, does not even know who is underneath it. I wonder if this dress, the Prima Donna, in a way is everything there is. Will the person underneath the green dress disappear if the dress is taken off? I wonder, how can we understand the existential balance between the dress and the one who carries the dress?

Reading ‘Breaking Down the Wall’, another sentence comes to me. It is the father’s words: “*You become very watchful*”. It makes me think that this father really has a wish to see. But something is in the way. He wants to see—but it is not possible for him. His seeing meets a barrier—a wall. The sentence also makes me think of different ways of seeing. Being watchful makes me think of surveillance. It makes me think of the way a professional collects data by recording in sometimes rather objective ways. The word watchful also makes me think of responsibility. This father carries a great responsibility. It is essential, that he sees. But what is this father supposed to see? And what is it, that he needs to see, which he is not able to see? I am not sure he—or anyone else—knows. I sense despair—a sense that the daughter’s whole life is dependent on the seeing of her father. But he is not able to see, although desperately trying to.

The two narratives could be comprehended as narratives of a wish to be seen and a wish to see. In that way, the narratives carry a great sorrow as something seems to hinder the possibility of seeing. The father desperately wants to see. The woman underneath the dress so desperately wants to be seen. But no one in these narratives knows how to see. No one knows what should be seen. No one really knows how to see beyond the wall or behind the dress.

In the late works of the French philosopher, Maurice Merleau-Ponty, seeing is in focus. In his little book ‘L’oeil et l’esprit’ (in this article the Norwegian translation ‘Øyet og ånden’ [45] has been used), Merleau-Ponty is preoccupied with the way painters like Paul Cezanne or Paul Klee see, as he thinks their seeing is exemplary for the way researchers and human beings as such, ought to see. How does a painter see the mountains, that he so convincingly paints, Merleau-Ponty wonders. And he answers that the painter sees much more than “visual facts” [45, p. 25]. The painter sees much more than a 1:1 reproduction of the landscape in front of him. The painter’s seeing is not one-dimensional, but it opens into a living world of several dimensions. The painter’s eye has a sense of and openness towards what Merleau-Ponty describes as ‘the secretive’ in our landscapes and in our lives as such. The painter’s eye opens into a visibility of a second order [45, p. 19].

I wonder if it is a seeing of a ‘second order’, that is missing in the two narratives. I question whether the two narratives, in the light of Merleau-Ponty’s thinking, could be comprehended as narratives of a seeing that has become too one-dimensional, and, at the same time, narratives pointing towards a longing to be able to see ‘more’—more than the visual facts, more than what is up front. If so, a further question might be, how the painter sees this ‘more’? How does the painter see beyond the visual facts? Merleau-Ponty’s answer is that the painter’s eye is both receiving and questioning.

The painter’s eye receives impressions of light, properties, colours, depth and so forth with great openness. It is, in a way, an unprotected eye receiving whatever is coming. And these impressions do something with the eye. The eye is set in motion by its seeing [45, p. 23]. The eye changes with what it sees. The naked eye is overwhelmed and formed by the landscape. Moreover, this formation sets the whole painter in motion. It changes his perspectives, and it leads his hands to paint. The receiving eye has consequences for the one who sees. It’s not free of charge to see in a receiving way.

At the same time, the painter’s eye is a questioning eye. By that Merleau-Ponty describes the painter’s eye as exploratory. The kind of exploration Merleau-Ponty writes about is not an exploration from the outside. It is *not* an objective or untouched exploration. The questioning eye questions the landscape from the inside. The questioning eye asks the landscape (the mountain for instance) to give away its secrets of being. It is a questioning, not from a ‘knower’ but from an ‘ignorant’. From one who does not know, but so much longs to see and thereby sense just a bit more [45, pp. 71–73].

Reading the above narratives in the light of Merleau-Ponty, I wonder if his different kinds of seeing can be informative. I wonder if the barriers to seeing, described by words such as wall and dress, are a barrier to see ‘more’ than visual facts. A barrier to see more than the directly visible in the daily life of this family. Maybe the wall and the dress are the family’s way of putting into words, that they so much want to see and to be seen by one another in different ways, than they are used to.

#### Bodil

Bodil is a nurse and holds a PhD in health sciences. She works as a professor at UiT The Arctic University of Norway. Bodil’s expertise is in narrative inquiry. She has conducted several narrative studies and has published a textbook and edited a collection about narrative research. In recent years, her main interest has been in the further development of the concept and theoretical foundation of narrative care. She has no clinical experience with EDs.

I was honoured when Berit invited me to contribute to this article. And I was moved when I listened to Berit reading the narrative about the Playmobil figure and the narrative about the wall, and later, when I read and re-read the two narratives. Berit invited me to contribute with an unpacking of the two narratives from the perspective of a narrative inquirer. However, narrative inquiry is the study of experience over time and in contexts [46], and essential to narrative inquiry is the “collaboration between researcher and participants, over time, in a place or series of places, and in social interaction with milieus” [46, p. 20]. Since I had not been involved in Berit’s fieldwork, I had not had the opportunity to engage with the young woman and her family over time and in different contexts. All I had, were two narratives about a Playmobil figure and a wall—the first, narrated in Berit’s fieldnotes and the second, narrated by the young woman’s father in an interview with Berit. Hence, by accepting Berit’s generous invitation, I had put myself in a position in which I felt somewhat uncomfortable and alienated—a position requiring that I “use stories as data; view narrative and story as representational form: […] or treat stories as the phenomena under study” [47, p. 575], that is, research practices with which I do not identify. To me, narrative inquiry is not about scrutinising narratives and subjecting them to analysis. Nonetheless, the two narratives stayed with me, and I continued to think about and with them.

As a narrative inquirer, I consider narrative neither as a representation of experience nor as a metaphor for ‘the real world’. Rather, for me, narrative is a way of experiencing and making sense of the world. I agree with Clandinin [48] that, as narrative inquirers, we “need to pay close attention to who we are in the inquiry and to understand that we, ourselves, are part of the storied landscapes we are studying” [48, p. 30]. And here, perhaps, lies one answer to why I could not let go of the Playmobil figure and the wall, despite my unease. I engaged with these narratives, not solely as a narrative inquirer, but also as a woman, a daughter, a mother, and a partner. And I read them as a person who has experience with an ED in early adulthood.

As a narrative inquirer, I cannot and will not speculate on the symbolism of the green dress and the wall. Rather, my reflections on the two narratives revolve around the two interconnected concepts of ‘world travelling’ and ‘playfulness’. In particular, two sentences spoke to me: *I’m the only one with the key to the solution* and *You don’t start to climb the wall, because you don’t know what awaits you on the other side*. Both these sentences speak to me about the absence of, and, perhaps, longing for a common space to explore. Lugones [49] has written insightfully about world travelling. By metaphorically travelling to the other’s world, we are allowed to identify with the other “because by travelling to [the other’s] ‘world’ we can understand what it is to be them and what it is to be ourselves in their eyes” [49, p. 17]. Integral to world travelling is playfulness, described by Lugones [49] as an openness to uncertainty and to surprise that acknowledges that we “are not fixed in particular constructions of ourselves” [49, p. 16]. The father’s statement is a strong reminder of the uncertainty inherent in all relationships—*you don’t know what awaits you on the other side*. By climbing the wall, you risk not only facing that your constructions of the other may be wrong, you also risk facing the other’s constructions of yourself.

Living with an ED is an embodied experience. And I know from experience, that it is also a lonely experience—expressed in the young woman’s statement, *I’m the only one with the key to the solution*. The statement might also embody the heavy burden of knowing that your illness not only affects your own life but also the lives of the people close to you. Hydén [50] has noted that embodied experiences “reside in the actual ways the body moves, the voice or artefacts that are used” [50, p. 235]. Bodily communicative events are perhaps even more salient when the capacity to compose coherent stories is compromised. The elements of ‘playfulness’, imagination, and improvisation are evident in the narrative about the Playmobil figure. Perhaps, was the Playmobil figure exercise an opportunity for the young woman to explore experiences that resisted verbal expression? The exercise may be understood as a narrative practice that allowed the participants to move past fixed stories and experiences, and to collaboratively explore experiences that were difficult or impossible to verbalise.

As stated above, the aim of the MFT programme is not primarily to cure the ED, but rather to support communication and interaction in the families involved. From my perspective as a narrative inquirer, I would frame this aim as ‘shaping new stories to live by’ [51]. It is my sincere hope that the MFT programme, through world travelling and playfulness, gives families the opportunity to co-compose spaces in which they can retell and re-live their experiences in ways that allow them to become otherwise [52].

## Discussion

This article is a response to Conti and colleagues’ call for relational metaphors to supplement the traditional medical / psychological diagnostic language used to describe the life experiences and complex emotions of people affected by an ED [1]. We were inspired by their call for “…a different kind of listening; an attention to metaphors that we do not know, plot lines we cannot follow and problems we have not fully recognized” [1, p. 3].

In this article, four authors from different professional, theoretical and methodological backgrounds have taken the same two narratives as our point of departure for our individual and collaborative unpackings. Our aim was twofold: first, we wished to explore the inherent potential of multiple perspectives in understanding experiences of living with an ED. Second, we wished to explore the potential of engaging in dialogue between different perspectives. We concur with Conti et al. who argued that new types of stories will shift our understandings and shape and change our research and therapy practices [1, p. 5]. We further argue that inquiring into “the new types of stories” from multiple perspectives and in dialogue will further enrich both clinical practice and research.

Notably, our individual unpackings focused on quite different aspects of the two narratives. Siri’s unpacking focused on the strength and pride of the Playmobil figure (the Prima Donna), seeing the figure as an artistic expression in a language different from the spoken word (*“Maybe the whole point is that she has tried to convey something with a different type of language”).* Berit’s unpacking focused on the experience of isolation, and the necessity of balancing openness and the need for boundaries (*“I find the narrative about the wall to be a beautiful and powerful metaphor, speaking on many levels of the father’s and family’s experience of loneliness, isolation, and struggles to move forward”*). Sine’s unpacking dwelled on the wish to be seen and the wish to see (*“The dress makes a silent cry: Watch me, it says” “The father’s words ‘You become very watchful’ makes me think, that this father has a wish to see.”*). And, finally, Bodil lingered with the lonely and uncertain experience of living with an ED and the absence of a common space to explore, expressed in the two narratives *(“In particular, two sentences spoke to me: I’m the only one with the key to the solution and You don’t start to climb the wall, because you don’t know what awaits you on the other side”)*. Reading our individual unpackings, and focusing on the differences between them, we were reminded that our understandings are always partial and unfinalised. None of our individual unpackings represents ‘the truth’. Rather, through dialogue between our respective perspectives, we have strived towards ‘thick description’ [39] and deeper and more nuanced understandings. As researchers and clinicians, we are not neutral recipients of people’s stories. Rather, our understandings are framed and shaped by our personal and professional backgrounds. In that regard, we agree with Josselson [53] who noted that, “We need to say who we are as interpreters who bring our own subjectivity to the topic or people we are writing about” [53, p. 49]. Moreover, we are aware that our individual unpackings are not interpretations of the persons’ lives. Rather, they represent four possible openings to learning and further understanding of lived experiences of living with an ED in the family. As Gadamer noted, every understanding is a draft, an opening to a possible world in which we can gain further and deeper insight. Understanding is, in that sense, always preliminary [38]. Such ever-growing understandings might not be about this family in particular, but about human lives and relationships in general (such as the balancing between openness and the need for boundaries, the longing to be seen and the wish to see, and the need for common spaces to explore).

Through the process of writing our own and reading each other’s unpackings we were reminded that our understandings of phenomena are always partial. And through the continued dialogue, we were reminded of the importance of encountering people’s narratives with humility, curiosity and wonder because things could always be otherwise. None of us claimed to have found ‘the truth’. Rather, we acknowledged that ‘truths’ about human lives may be multiple and complex. Truths are both contextual, temporal and relational, as are the truths of living with an ED in this specific family. At the same time, truths can carry traces of universality, when unpackings open into human life as such. Phenomenologically, truths are connected to “the possibility of plausible insight which brings us in more direct contact with the world” [54, p. 37]. This is not the same as claiming that ‘anything goes’—a position of total relativism. As Geertz notes, there are differences between good and less good interpretations. “A good interpretation of anything—a poem, a person, a history, a ritual, an institution, a society—takes us into the heart of that of which it is the interpretation. When it does not do that, but leads us instead somewhere else—into an admiration of its own elegance, of its author’s cleverness, or of the beauties of Euclidean order—it may have its intrinsic charms; but it is something else…” [39, p. 18]. Our unpackings became ‘plausible’ because our dialogue required that we made our theoretical and methodological presuppositions explicit, not giving a priori one particular perspective interpretive authority [53, p. 49] and because we all tried to go into, what we experienced as ‘the heart of things’. We read Conti and colleagues’ article [1] as a call not to give one perspective—the medical/psychological language—a priori interpretive authority, but rather keep other possible understandings of EDs open through the introduction of and dialogue between different metaphors, stories and perspectives.

### Methodological considerations

In this article, the family’s voices are only represented through the two narratives. Neither the young woman nor her family were invited to do their own unpacking of the two narratives or comment on our unpackings as they had previously declined an invitation to participate in further research. Including the woman and her family in the writing of this article would have contributed to further nuance and depth in the understanding of experiences with living with an ED. This article should be read bearing that in mind.

## Conclusion

Although there is a substantial body of research into the experiences of living with an ED, much remains unknown. In this article, we have argued for the importance of engaging with multiple perspectives and dialogue, and we sincerely believe it is necessary to ‘break down the wall’ between clinicians and researchers from different traditions and paradigms. Caring for people living with an ED calls for multiple perspectives and dialogue. In order to co-compose new and deeper understandings, we need to challenge the dominant narratives about EDs through engaging with the narratives of those living with an ED with curiosity, openness and willingness to learn.

## Data Availability

The dataset analysed during the current study are available from the corresponding author on reasonable request.

## Abbreviations

AN: Anorexia nervosa
BN: Bulimia nervosa
ED: Eating disorder
MFT: Multifamily therapy
RESSP: Regional Centre for Eating Disorders
